# Severe Pneumonia Caused by Toxigenic *Corynebacterium ulcerans* Infection, Japan

**DOI:** 10.3201/eid2403.171837

**Published:** 2018-03

**Authors:** Ikkoh Yasuda, Hisayo Matsuyama, Tomoko Ishifuji, Yoshiro Yamashita, Masahiro Takaki, Konosuke Morimoto, Motohiro Sekino, Katsunori Yanagihara, Tatsuya Fujii, Masaaki Iwaki, Akihiko Yamamoto, Koya Ariyoshi, Takeshi Tanaka

**Affiliations:** Nagasaki University Hospital, Nagasaki, Japan (I. Yasuda, Y. Yamashita, M. Takaki, K. Morimoto, M. Sekino, K. Yanagihara, K. Ariyoshi, T. Tanaka);; Kawakita General Hospital, Suginami, Tokyo, Japan (H. Matsuyama, T. Ishifuji, T. Fujii);; National Institute of Infectious Diseases, Shinjuku, Tokyo (M. Iwaki, A. Yamamoto)

**Keywords:** *Corynebacterium ulcerans*, pneumonia, diphtheria, zoonoses, pseudomembrane, bacteria, Japan

## Abstract

*Corynebacterium ulcerans* infection was recently recognized as a zoonosis. We present 2 cases of severe pneumonia complicated by diffuse pseudomembrane formation on the bronchus caused by *C. ulcerans*–producing diphtheria toxin. Our purpose is to alert medical professionals to the virulence of *Corynebacterium* species other than *C. diphtheriae*.

The well-known *Corynebacterium* species is *C. diphtheriae,* which causes diphtheria. *C. ulcerans* has recently been identified as a causative agent of zoonotic infection and isolated from a wide range of domestic and wild animals (*1*). The bacterium has increasingly been reported in western Europe (*2*,*3*), and the number of reported cases of respiratory infection with *C. ulcerans* has been increasing (*4*–*6*). Certain strains produce diphtheria toxin and can cause a serious condition similar to diphtheria infection (*7*). This infection has a predilection for the upper respiratory tract; pneumonia caused by *C. ulcerans* is rare but can be fatal (*8*). We describe 2 cases of severe pneumonia caused by *C. ulcerans* with confirmed production of diphtheria toxin.

## The Study

**Figure 1:**
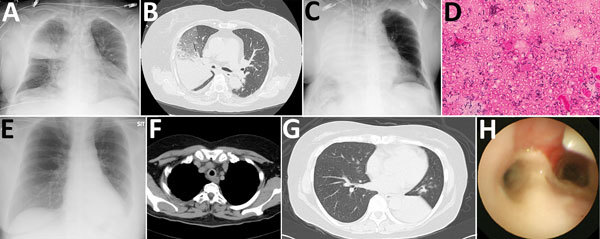
Radiological imaging, sputum smear finding, and endobronchial image of 2 case-patients with severe pneumonia caused by *Corynebacterium ulcerans* infection, Japan. Case-patient 1: A) Atelectasis in middle lung field at admission; B) consolidation and atelectasis in right upper lobe; C) rapid development of diffuse atelectasis; D) gram-positive rods in all fields of endotracheal aspiration sputum sample (original magnification x1,000). Case-patient 2: E) Infiltrates in left lower lung field; F) thickened tracheal membrane; G) Atelectasis of left lower lobe; H) pseudomembrane formation on trachea and bronchus.

Case-patient 1, a 67-year-old woman with hepatocellular carcinoma, hypertrophic obstructive cardiomyopathy, and type 2 diabetes mellitus, came to Nagasaki University Hospital with severe dyspnea. She kept a cat and a dog in her house and also had close contact with stray cats in her neighborhood. Four days previously, she had noticed pharyngeal pain, cough, and nasal discharge. The dyspnea developed 2 days before the admission. The patient’s body temperature was 38.0°C, blood pressure was 110/82 mm Hg, and respiratory rate was 32 breaths/min; oxygen saturation was 56% on room air. We gave her 10 L of oxygen via face mask with reservoir bag (Table 1). We observed no patches of exudates over the pharynx. Chest radiography showed right lung infiltrates (Figure 1, panel A). Computed tomography (CT) revealed consolidation, ground-glass opacity in the right upper and lower lobe, and thickened right main bronchus wall (Figure 1, panel B). Due to the rapid progression of respiratory failure and shock on the day of admission, we admitted the patient to intensive care and intubated her. The right lung collapsed within a few hours (Figure 1, panel C). Bronchoscopic observation revealed obstruction of the right main bronchus with massive white and yellowish exudate (pseudomembrane). We removed part of the pseudomembrane using forceps, reopening the right main bronchus and improving oxygenation. We observed club-shaped gram-positive rods on Gram staining of the exudate (Figure 1, panel D) and isolated *C. ulcerans*. We initiated intravenous vancomycin, tazobactam/piperacillin, and azithromycin (AZM) empirically, followed by sulbactam/ampicillin (SBT/ABPC). The bacterium was sensitive to ampicillin, gentamicin, erythromycin, and levofloxacin. The patient responded well to the treatment without administration of diphtheria antitoxin and was extubated on day 5 after admission. We discharged her ambulatory on day 30 after admission.

**Table 1:** Laboratory data at admission for 2 case-patients with severe pneumonia, Japan

Test	Case-patient 1	Case-patient 2
Leukocytes, cells/mm^3^	13,800	23,200
Neutrophils, %	85	90
Lymphocytes, %	4	4.2
Erythrocytes, × 10^4^/µL	448	428
Hemoglobin, g/dL	12.6	13.1
Hematocrit, %	41.2	38.4
Platelets, × 10^4^/µL	24	26.2
Total bilirubin, mg/dL	0.1	0.6
Aspartate aminotransferase, IU/L	26	18
Alanine aminotransferase, IU/L	20	14
Lactate dehydrogenase, IU/L	238	249
Blood urea nitrogen, mg/dL	43	11
Creatinine, mg/dL	1.31	0.7
C-reactive protein, mg/dL	13.58	31.8
Administered oxygen	Mask, 10 L oxygen	Nasal, 2 L oxygen
Arterial blood gas levels		
PaCO_2_, mm Hg*	56.1	36.7
PaO_2,_ mm Hg†	69.1	72.2
pH	7.263	7.45
Bicarbonate, mmol/L	24.5	25
Base excess, mmol/L	−2.7	1.3
Lactate, mmol/L	0.9	Not tested

*Arterial carbon dioxide partial pressure †Arterial oxygen partial pressure

Case-patient 2, a 66-year-old woman with borderline diabetes, was admitted to Kawakita General Hospital in Tokyo for febrile dyspnea. She kept 3 cats in her house and had close contact with them. Throat pain and cough had developed 10 days before the admission. The patient’s body temperature was 37.6°C, blood pressure was 135/78 mm Hg, and respiratory rate was 16 breaths/min; oxygen saturation was 87% on room air (Table 1). There was no lymphadenopathy or white spots on the pharynx. Chest radiography showed left lung infiltrates (Figure 1, panel E). We observed tracheal and bronchial wall thickening causing occlusive atelectasis of the left lower lobe (Figure 1, panels F, G). We administered SBT/ABPC and AZM. Because of rapidly exacerbating respiratory distress on the second hospital day, we intubated the patient. We administered SBT/ABPC continuously but replaced AZM with ciprofloxacin (CPFX). Bronchoscopy revealed white-yellowish exudate covering the whole left main bronchus and narrowing the lumen (Figure 1, panel H). We found massive gram-positive rods on Gram staining of the exudate. *C. ulcerans* sensitive to ampicillin, gentamicin, erythromycin, and levofloxacin was isolated. The patient responded to the treatment without administration of diphtheria antitoxin and was extubated on the 12th hospital day. The wall thickness and atelectasis improved. Consolidation and ground glass opacity, compatible with organizing pneumonia, persisted and improved gradually. We discharged the patient with no serious sequelae on the 35th day after admission.

We performed environmental surveillance for patients’ companion animals and their belongings. We conducted swab sample collections for culture at the sites where the cats were housed and performed sampling from animals with their owners’ informed consent and in their presence. We performed toxigenicity analysis and determined antidiphtheria toxin neutralization titers by Vero cell cytotoxicity assay, as described by Katsukawa et al. (*9**,**10*). We performed ribotyping of isolated strains and conducted *rpoB* gene sequencing with the primers C2700F and C3130R as described by Khamis et al. (*11*). 

Case-patient 1 had fed her pet cat and dog and 1 stray cat. At the patient’s house, we detected *C. ulcerans* in the feeding tray, on the surface of the furniture on which the cat lay, on the cat’s blanket, and on the telephone receiver. Samples from stray cats were not available. For case-patient 2, we treated her 2 cats that tested positive for *C. ulcerans* with erythromycin. Isolates from case-patient 1, case-patient 2, and case-patient 2’s cats were all toxigenic (Table 2). We analyzed antitoxin titers from case-patients 1 and 2. Case-patient 1 showed a nonprotective level of antitoxin (<0.01 IU/mL) on day 12 after onset; however, her husband showed a protective level (>0.1 IU/mL). Case-patient 2 showed a protective level 1.5 months after onset (334 IU/mL). Case-patient 1 received an additional vaccination with diphtheria toxoid, but case-patient 2 did not. 

**Table 2:** Characteristics of *Corynebacterium ulcerans* isolates identified from swabs of 2 case-patients with severe pneumonia and their environments, Japan*

Sample	Culture	*rpoB* gene	*tox* gene	Vero cell cytotoxicity
Case-patient 1				
Patient sputum	Positive	*C. ulcerans*	+	+
Husband throat swab	Negative			
Cat throat swab	Negative			
Swab from cat’s feeding tray	Positive	*C. ulcerans*	+	Not tested
Swab from cat’s blanket	Positive	*C. ulcerans*	+	Not tested
Swab from telephone receiver	Positive	*C. ulcerans*	+	Not tested
Case-patient 2				
Patient sputum	Positive	*C. ulcerans*	+	+
Cat 1 throat swab	Positive	*C. ulcerans*	+	+
Cat 2 throat swab	Positive	*C. ulcerans*	+	+
Cat 3 throat swab	Negative			

*Detectable by PCR (*tox* gene) or Vero cell cytotoxicity assay. +, positive.

**Figure 2:**
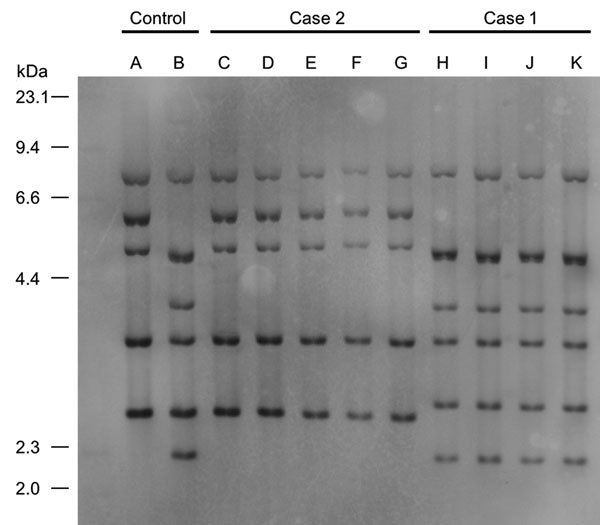
Figure 2. Ribotyping tests for isolates from 2 case-patients with severe pneumonia caused by *Corynebacterium ulcerans* infection, Japan. Case-patient 1 isolate was categorized as R3 group and case-patient 2 isolate as R1 group. A, 0102 (R1 group); B, 0211 (R4 group); C, isolate from case-patient 2’s exudate on bronchus; D, isolate from cat 1’s throat in case 2; E, isolate from cat 2’s nasal cavity in case 2; F, isolate from cat 2’s throat in case 2; G, isolate from cat 2’s conjunctiva in case 2; H, isolate from case-patient 1’s exudate on bronchus; I, isolate from cat’s feeding tray in case-patient 1’s house; J, isolate from cat’s blanket in case-patient 1’s house; K, isolate from telephone receiver in case-patient 1’s house.

Ribotyping of *C. ulcerans* isolates from the cases matched that of the cats and cat environment in their households. We categorized ribotyping for isolates from case-patient 1 as R3 group and for case-patient 2 as R1 group (Figure 2).

## Conclusions

The mode of transmission of *C. ulcerans* to humans via infected companion animals was well documented in our cases. We demonstrated diphtheria toxin from both case-patients and isolation of *C. ulcerans* from case-patients and their companion animals. An immunization program for diphtheria in Japan started at the time of these case-patients’ births; therefore, their immunization histories for diphtheria were uncertain. The antitoxin level of case-patient 1 was considered nonprotective according to World Health Organization guidelines, whereas her husband’s antitoxin level was considered fully protective (*12*). This finding may explain why her husband did not have the same severe condition develop. Although case-patient 1 received the diphtheria vaccine after discharge, little is known about the efficacy of the diphtheria toxoid vaccine in preventing *C. ulcerans* infection, and recommendation of its prophylaxis with diphtheria antitoxin remains controversial (*13*–*15*).

Both cases showed similar patterns of acute respiratory failure. Thickened bronchial wall and pseudomembrane formation caused airway obstruction and severe atelectasis, leading to rapid progress of respiratory insufficiency. Release of the obstruction improved oxygenation dramatically. We considered acute atelectasis development and not damage to lung parenchyma, which is usually seen in severe pneumonia, to be the main cause of respiratory failure. 

Facilities in which diphtheria toxin analysis is available remain limited. In some areas where reporting systems for *C. ulcerans* have not been introduced, accurate diagnosis is often missed. When Gram stain findings and endotracheal appearance point to infection, clinicians must pay careful attention to respiratory management for airway obstruction.

In summary, severe pneumonia caused by *C. ulcerans* is uncommon but life-threatening. It is important to recognize that *C. ulcerans* can be pathogenic in humans and that outcomes could be fatal.
